# Filter Selection for Optimizing the Spectral Sensitivity of Broadband Multispectral Cameras Based on Maximum Linear Independence

**DOI:** 10.3390/s18051455

**Published:** 2018-05-07

**Authors:** Sui-Xian Li

**Affiliations:** 1 Flying College, Binzhou University, Binzhou 256600, China; leesx_72@163.com; Tel.: +86-186-0089-0892; 2 Aeronautic Information Technology Research Center, Binzhou University, Binzhou 256600, China

**Keywords:** spectral sensitivity optimization, filter design, multispectral imaging, broadband filter set, color reproduction, spectral reconstruction, sensor

## Abstract

Previous research has shown that the effectiveness of selecting filter sets from among a large set of commercial broadband filters by a vector analysis method based on maximum linear independence (MLI). However, the traditional MLI approach is suboptimal due to the need to predefine the first filter of the selected filter set to be the maximum ℓ_2_ norm among all available filters. An exhaustive imaging simulation with every single filter serving as the first filter is conducted to investigate the features of the most competent filter set. From the simulation, the characteristics of the most competent filter set are discovered. Besides minimization of the condition number, the geometric features of the best-performed filter set comprise a distinct transmittance peak along the wavelength axis of the first filter, a generally uniform distribution for the peaks of the filters and substantial overlaps of the transmittance curves of the adjacent filters. Therefore, the best-performed filter sets can be recognized intuitively by simple vector analysis and just a few experimental verifications. A practical two-step framework for selecting optimal filter set is recommended, which guarantees a significant enhancement of the performance of the systems. This work should be useful for optimizing the spectral sensitivity of broadband multispectral imaging sensors.

## 1. Introduction

Multispectral imaging refers to imaging with more than three to several tens of narrowband or broadband spectral channels. Since the nineties, multispectral color imaging involving spectrally sampling by broadband absorption filters has been explored in the multispectral imaging community [1,2,3,4,5,6]. Broadband multispectral imaging takes advantage over narrow multispectral imaging in reconstructing smooth spectra of imaging scenes using a far fewer number of spectral measurements [7], therefore it can significantly reduce the complexity of the needed hardware. With an equal number of spectral channels, moreover, because the broadband techniques preserve more spectral features such as transmission or absorption peaks than narrow multispectral imaging [7,8], broadband multispectral imaging should be more promising to reconstruct spectral information of imaging scenes. In principle, a broadband multispectral camera cannot acquire the spectral samples at each wavelength directly at the data acquisition stage as narrowband ones; it takes spectral images at the expense of extensive post-processing under the condition that the spectral sensitivity of the system is optimized. Therefore, optimizing the spectral sensitivity is crucial for setting up a broadband multispectral camera. 

There are reports about optimizing the spectral sensitivity of multispectral camera by selecting filters among a larger number of commercial filters [3,4,5,6,7,9,10,11,12], varying the spectrum distribution of light source using a large number of modulatable LEDs [13] or properly designing the response curve of spectral filter array (SFA) [14,15] and color filter array (CFA) sensors [16,17]. Among them, selecting a filter set from among a large set of commercial filters is an efficient and cheaper way to set up a multispectral camera [3,4,5,6,7,9,10,11,12]. There are two kinds of algorithms for filter selection. One is the filter vector analyzing method (FVAM). The FVAM is used for selecting a filter set directly from a large number of filters by considering the mathematical features of the filters without considering other imaging parameters such as the spectrum distribution (SPD) of illuminants, spectral sensitivity of the camera, the characteristics of imaging scene, etc. [3,7,9]. The other is the systematic recursion method (SRM), which searches exhaustively for an optimal filter set among all the combinations of a commercial available filter collections in terms of the results of spectrometric and/or colorimetric recovery [4,5,6,10,12]. The SRM is time-consuming because it requires a large amount of data acquisition and reconstruction, while the FVAM is more straightforward and can be conducted only by simple vector analysis of the filters. Among all the FVAMs, maximum linearity independence (MLI) performs better [3]. Hardeberg used the MLI method to select spectral training set for the first time [10], whereafter Li applied it to filter selection for optimizing the spectral sensitivity of broadband multispectral cameras [11]. The insight of the MLI method is the transmittances matrix of the selected filter set featuring a minimum condition number. The MLI method was verified to outperform other vector analysis methods such as maximizing the orthogonality in the vector space of the transmittances of broadband filters [3,11]. However, the limitation of the tradition MLI method is defined by the $\ell_{2}$ norm of the transmittance vector of the first filter being the maximum among all those of the filters to be selected. The traditional MLI method can assure the highest signal to noise ratio (SNR) for the response of the first channel of multispectral camera, however it hard to satisfy the optimization for the overall system, which will be verified in the following sections.

To the best of our knowledge, there has been no further investigation into the MLI method for broadband filter selection. In the following sections, the MLI method is investigated by varying the first filter through simulation of spectral imaging. From the analysis to best-performed filter sets, the traditional MLI filter selection method will be reformed for optimizing the spectral sensitivity of the broadband multispectral camera without considering other imaging parameters, except for the spectral transmittances of the filters. 

The article is organized as follows: The MLI filter selection method is introduced first in Section 2. Then an experimental simulation is described in Section 3, the features of the best-performing filter set from the experimental simulation results are abstracted in Section 4, and the generalization of the results of the article is discussed in Section 5. Finally, the conclusions are drawn in Section 6.

## 2. MLI Filter Selection Method

The MLI method follows the rules of the FVAM. The FVAM requires selecting an optimal filter set only by filter vector analysis. The characteristics of the optimal filter set are defined by some metrics describing the vectors of the filter set. These matrices are extracted from the vectors of the best-performing filter set, which are selected according to their performance in terms of color reproduction or spectral reconstruction [1,7,9]. The original proposal of MLI used in multispectral color imaging is relevant to training set selection. The objective is to seek a minimum number of spectral samples in a large set of collection for the most representative subset [10]. From the perspective of linear algebra, it means the selected sample subset span is as uniform as possible in the overall spectral space.

In order to describe the MLI method explicitly, we shall introduce the model of a multispectral camera beforehand. The multispectral camera model with a broadband filter set is this:

(1) $${\left[diag(L)\cdot diag(S)\cdot T\right]}^{T}R=C,$$

where the *diag*(.) denotes a diagonal matrix with elements of corresponding vector*,* and [.]^T^ denotes the transpose of a matrix.

(2) $$L={\left[l_{1},\ldots l_{i},\ldots ,l_{n}\right]}^{T}$$

is a column vector of light source spectral distribution with each elements corresponding to a wavelength sample.

(3) $$S={\left[s_{1},\ldots s_{i},\ldots ,s_{n}\right]}^{T}$$

is a column vector of spectral sensitivity of imaging sensor;

(4) $$T=\left[T_{1},\ldots ,T_{i},\ldots T_{m}\right]$$

is a matrix of spectral distribution of filter transmittances with *m* spectral sensing channels, each column of which is a transmittance vector of a corresponding filter corresponding with *n* wavelength sample; R and C are column vector of reflectance and response of camera sensor respectively. Let:

(5) $$\Phi ={\left[diag\left(L\right)diag\left(S\right)T\right]}^{T},$$

we can see the camera model describes a linear transform from **R** to **C** from Equations (1) and (5). From the above model, **Φ** should be an orthogonal matrix if the **R** can be precisely reconstructed theoretically if we ignore the noise. However, as previous investigated, the traditional MLI method is more practical than maximizing the orthogonality of **Φ**, owing to the fact it is constrained by the physics of the camera parameters [3,11]. 

The aim of the traditional MLI method algorithm is to find a filter set of *M* channels with minimum condition number from the transmittance vectors of all available filters. The physical constraints of the thickness and the transmittance of a filter must be taken into account. It would not be practical if the number of the filters stacked up to form a filter combination were larger than 2 due to larger thickness and smaller transmitted energy of rays. Therefore, the total number of the available transmittance vectors *K* should be:

(6) $$K=N+\left(\begin{array}{l} N\\ 2 \end{array}\right)=N+\frac{N!}{2!(N-2)!}$$

which comprise single filters and combinations of two filters, where *N* denotes the number of original transmittance vectors. The transmittance vector of the combination can be derived by multiplying each pair of the corresponding coordinates of the transmittance vectors two single filters.

The traditional MLI algorithm is detailed in pseudocode in Algorithm 1 as follows. Note that we define the transmittance vector matrix of the single chip filter collection as $T_{original}=[T_{o}^{1}\;...\;T_{o}^{i}\;...\;T_{o}^{N}]$, where each of the columns is a transmittance vector of a filter; the collection of the total number *K* of the available transmittance vectors are denoted $T_{\mathrm{int}egral}=[T_{i}^{1}\;...\;T_{i}^{i}\;...\;T_{i}^{K}]$. 

**Algorithm 1** Traditional MLI algorithm**Input** *S* = 1; *M*;*N*; $T_{original}=[T_{o}^{1}\;...\;T_{o}^{i}\;...\;T_{o}^{N}]$*;* **Procedure** (1) Computer $T_{\mathrm{int}egral}=[T_{i}^{1}\;...\;T_{i}^{i}\;...\;T_{i}^{K}]$ (2) Computer the $\ell_{2}$ norm set $\left\{\left\|T_{}^{i}\right\|\right\}$ (3) IF S==1 (4) Find the objective filter $T_{Objective}^{s}=T^{k},\mathrm{subject}\;\mathrm{to}\;\underset{i=1,2,...,K-s+1}{k=\arg\max}\left\|T^{i}\right\|$ (5) END IF (6) FOR *S* = 2:*M* (7) Delete $T_{objective}^{s-1}$ from $T_{\mathrm{int}egral}$ (8) Find $T_{objective}^{s}=T^{j},\mathrm{subject}\;\mathrm{to}\;\underset{j=1,2,...,K-s+1}{j=\arg\min cond}[T_{Objective},T_{\mathrm{Integer}al}^{j}]$ (9) END FOR (10) $T=T_{objective}^{M}$ **Output** The filter set T selected by MLI with M spectral channels.

From the traditional MLI algorithm above, we can see that the first selected filter is supposed to be the one with the maximum $\ell_{2}$ norm of the corresponding vector. This would lead to the highest signal to noise ratio in the first channel as expected. However, it does not mean that the first channel with the highest signal to noise ratio is necessarily the spectrally optimized one for the broadband multispectral camera. The rationality of this statement will be validated in Section 4.2.2, where it will be shown that the best-performing filter set is often not the traditional first filter, which has the maximum $\ell_{2}$ norm. Therefore, in order to improve the traditional MLI algorithm, it is necessary to investigate the performance of all the possible filter sets by varying the first filters. The straightforward aim is to find the features of the best-performing filter sets from the experimental results.

## 3. Experimental Simulation

### 3.1. Datasets

**Figure 1:**
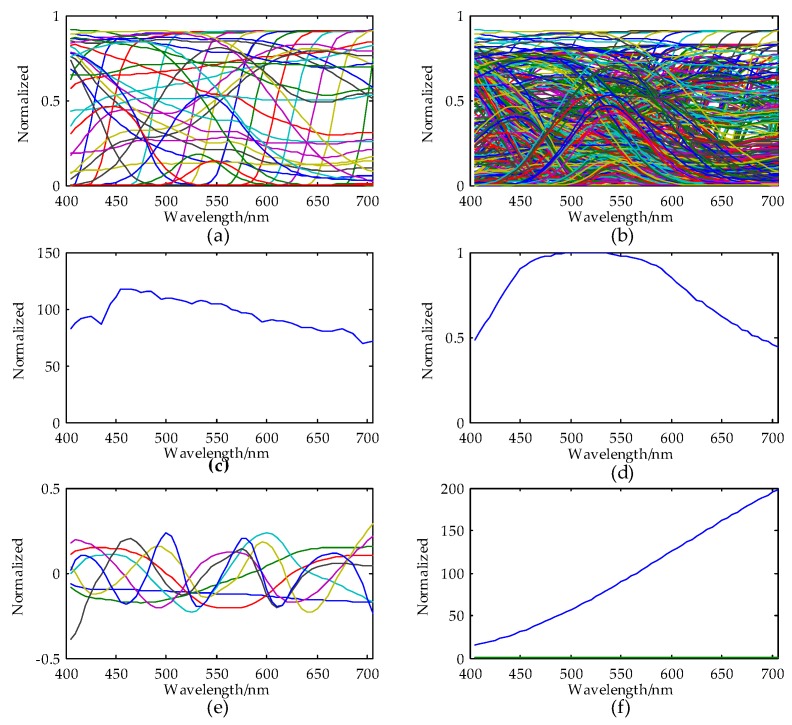

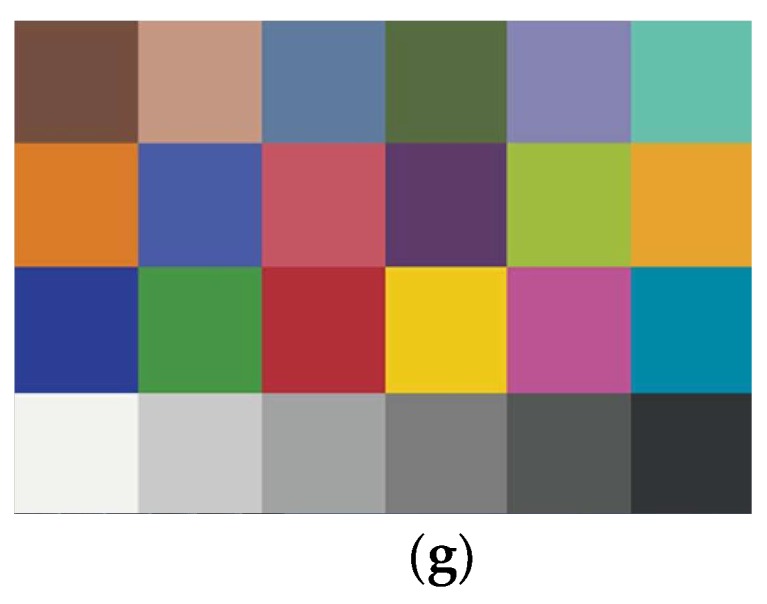
The data sets used for simulation are visualized above: (**a**) transmittances of 45 single filters; (**b**) all 1035 transmittances of single filters and two single filter combinations; (**c**) camera sensitivity of a Basler 302f camera sensor; (**d**) CIE standard illuminant D65; (**e**) The first eigenvectors of spectral data of the Macbeth ColorChecker; (**f**) CIE standard illuminant A and (**g**) color display of spectral data cubic of the Macbeth Color Checker.

In the next imaging simulation section, datasets comprise transmittances of filter sets, spectral sensitivity of camera, illuminant and spectral cubic image. The filter dataset is obtained from the Hoya Group color filter glass datasheet [18], which contains 45 transmittance vectors of the various single filters (see Figure 1a). In this case, the total number of the available transmittance vectors *K* is 1035 according to Equation (6). The 1035 vectors comprise transmittance vectors of the 45 single vectors and all the combinations of every two of them. 

The 1035 transmittance vectors are illustrated in Figure 1b. The CIE standard illuminant D65 and camera sensitivity of a Basler 302f sensor obtained from the manufacturer are displayed in Figure 1c,d. The spectral data cube contains 320 × 582 = 186,240 voxels, which is formed by 24 reflectance spectra derived from the measurements of the 24 patches of the Macbeth Color Checker measured by PR750; each patch of which comprises 80 × 97 voxels (see Figure 1g for its 2D display. The three “voxels” axes are the position coordinates for x,y and the spectral coordinate for reflectance. All the spectra contain 61 samples, respectively, in the wavelength range from 400–700 nm.

### 3.2. Imaging Simulation and Evaluation

Taking each of the 45 single filters for the first filter of the selected filter set, then other filters of the intended filter set are selected from the rest of 1034 filter transmittances according to step (4) to step (9) in the traditional MLI algorithm described above. Meanwhile, the corresponding condition number of the final selected filter set is recorded. To avoid ambiguity, the term “No. of filter” is defined as the serial number of the single 45 filters, and the “No. of filter set series” means a filter set series containing four to eight filter channels of which the first filter is from the sequential 45 filters in the following. For every selected filter set, the imaging simulation below comprises three steps. Firstly, we compute the camera response (CResponse) of the original reflectance ($R_{Original}$) by adding Gaussian noise ($N_{\mathrm{Gaussian}}$) according to Equation (7):

(7) CResponse=([diag(L)diag(S)T]TROriginal)T+NGaussian.

Secondly, a reconstructed spectrum (RRecounstruct) is computed according to Equation (8):

(8) RRecounstruct=ψ0[(ATA)-1AT]CResponse,

where $A={\left({\left[diag(L)diag(S)T\right]}^{T}R_{Original}\right)}^{T}\psi_{0}$; the part in the square blanket can be computed by the *matlab* function *pinv*(.), which denotes pseudoinverse of a matrix; and Ψ_0_ is a matrix which columns are the first *m* eigenvectors of the spectral reflectance of the Macbeth ColorChecker (*m* is the number of channels of the multispectral camera). More generally speaking, Ψ_0_ represents the a priori information of the reflectance of the imaging scene. Maloney and Wandell used the spectra reconstruction algorithm for the first time, in which the insight is making use of a linear approximation for the reflectance by the basis vectors of the a priori spectral training set [19,20]. Finally, we evaluate the performance of spectral reconstruction relevant to the selected filter sets.

To the best of our knowledge, a single metric is not capable of evaluating both the performance of spectral reconstruction and color reproduction in multispectral imaging. Therefore, several indices, such as PSNR, GFC, CIEDE2000 and MSE are often employed in the multispectral community [2,21,22] for evaluating the performances of the selected filter sets. Note that GFC, which means goodness of fit coefficient proposed by Romero et al., is a metric to measure the fitness of the estimated spectrum and the reference one [23]. Considering the relatively excellent results in the following simulation computed by the expression, GFC used in [2,22,23] are almost equal to 1, that is to say, losing its discrimination according to our former computation, the embedded Matlab function of *GoodnessofFit* [24] is adopted to compute GFC. The formula used is as follows:

(9) $${\mathrm{GFC}}_{\mathrm{NMSE}}=1-\frac{\left\|R-\hat{R}\right\|}{\left\|R-\mathrm{mean}\left(\hat{R}\right)\right\|},$$

where the cost function is normalized root mean square error (NMSE) of the estimated reflectance vector $\hat{R}$ and the reference reflectance vector R. The NMSE costs vary between –Inf. (bad fit) to 1 (perfect fit). Generally, the higher of PSNR, the more of GFC close to 1, and the more of CIEDE2000 and MSE approach to 0 are, the more optimal of the corresponding filter set is. 

In the simulation, we adopt five channel numbers of the multispectral camera; the numbers of these spectral channels are 4, 5, 6, 7 and 8, respectively. The level of additive Gaussian noise denoted by SNR is varied by the ten noise levels in dBs, where SNR∈{∞, 50, 47, 43, 40, 37, 33, 30, 27, 23}, and SNR = ∞ means there is no noise in the imaging system. The relationship of SNR and the noise variance σ is defined by:

(10) $$\sigma =10^{-\frac{\mathrm{SNR}}{10}}.$$

The noise is added by the *imnoise* Matlab function [21] to the response image R_esponse_ described in Equation (7). 

## 4. Data Process, Results and Analysis 

### 4.1. Data Process Methods

#### 4.1.1. Data Reduction

All of the 2250 groups of results from the imaging simulation are computed for investigation. Each group is relevant to a specific number of channels and a noise level, and contains four evaluation metrics that are PNSR, GFC, CIEDE2000 and MSE. 

**Figure 2:**
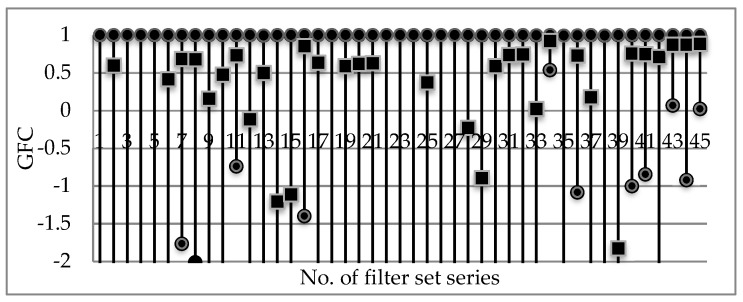
Overall performances of the 45 series of filter sets in terms of GFC, where the upper round marker denotes the maximum value; the middle square denotes the mean; and the lower round denotes the minimum (some of the minimums with negative value less than −2 are not be displayed).

**Figure 3:**
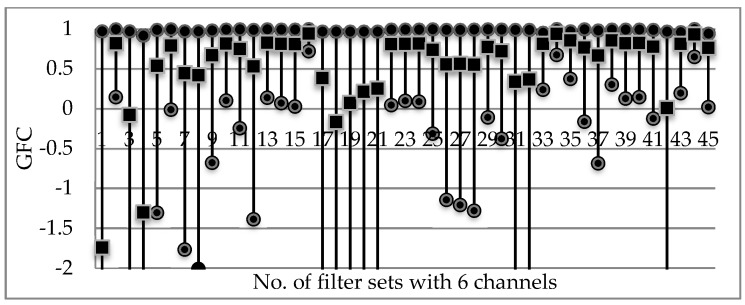
Overall performances of the 45 series of filter sets for 6 channels in terms of GFC, where the upper round marker denotes the maximum value; the middle square denotes the mean; and the lower round denotes the minimum (some of the minimums with negative value less than −2 are not be displayed).

Furthermore, we have computed the mean, maximum and minimum of all the 50 datasets (5 channel numbers × 10 noise levels) relevant to each one of the filter set series. Figure 2 shows the overall performances of the 45 series of filter sets in terms of GFCs, which are derived from average all the values regardless of the noise levels and the number of channels. We can see clearly that which of the series performs better intuitively, and the filter sets with specific channels and/or specific noise levels, as we can see in Figure 3 as an example. In this way, the best-performed filter set can be identified by one metric. However, it is a disputed question for a single competent metric evaluating both the performances of color reproduction and spectra reconstruction.

Efforts have been conducted to advance a single metric for evaluating both the performances of color reproduction and spectra reconstruction. Imai et al. concluded that there is no consensus metric that can been recommended as conclusively superior to others for all purposes [25]. López-Alvarez, et al. constructed a simple single-cost metric CSCM that combined several metrics at once, but it is designed for evaluating daylight spectra recovery by a simulated annealing algorithm [26]. In most cases, several evaluation metrics are adopted separately such as PNSR, GFC, CIEDE2000, MSE and so on [2,6,14,23,24,25,27,28]. Despite the fact the metrics are roughly consistent with each other, perceptible conflicts of such indices exist because of the differences in their corresponding definitions. 

Suppose a filter set has the maximum frequency from the best-performed filter sets collection in terms of different metrics, then we should conclude reasonably that the filter set is the best one. On the other hand, the best-performed filter sets in terms of different metrics are highly clustered in most of the cases, which can be seen from the numerical analysis in Section 4.1.2. Thus, the number of filter sets deserving further investigating should be greatly reduced. In the following subsections, we will investigate the top nine best-performing filter sets or series (accounting for 20% of the total number of filter sets) in terms of the four different metrics. The objective is to identify the best-performing filter sets based on the imaging simulation results.

#### 4.1.2. Data Sorting 

Table 1 list the results of the top 20% best-performing filter sets in terms of PSNR, GFC, MSE, CIEDE2000, where the data of PSNR and GFC are sorted in descending order, and those of MSE and CIEDE2000 are sorted in ascending order. All the data are derived from the corresponding averages of each metric, which include those of all the 10 noise levels and five channel numbers of the filter set series. 

**Figure 4:**
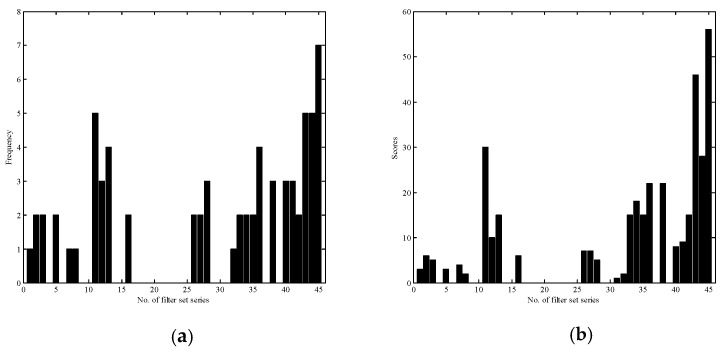
Statistics of the top 20% best-performed filter set series in terms of the four indices, PSNR, GFC, MSE and CIEDE2000: (**a**) frequency and (**b**) cumulative scores.

From Table 1, we can see the order of the sorted data relevant to each specific filter set series is not rigorously consistent; however, it shows that some of the filter sets appear frequently. This indicates as expected that if one metric of the performance of a filter set is better, others are often not too bad. For convenience, we count the frequency of each of the emerged filter set series. The results are shown in Figure 4a, where we can see the No. 45 has the largest frequency in the nine best-performing results, and No. 114344 ranked in the second place. Note that some of the filter sets fall outside the subset of 20% best-performing filter sets, therefore they give 0 frequency in Figure 4. 

Another applicable method to decide explicitly which filter set is more optimal is a score ranking method. The score ranking method is designed like this: let the first filter series in Table 1 have the highest score of 9, the second have 8 and so on, then we can get the cumulative scores of the filter sets. The results are shown in Figure 4b, from where we can see that the above No. 11, 43 and 44 set series, which are ranked in the same second place in Figure 4a, can be discriminated numerically. Consequently, if there is not just one filter set series sharing the same maximum frequency, the score ranking method should be irreplaceable.

### 4.2. Results

#### 4.2.1. Best-Performed Filter Sets

The cumulative scores of the top 20% best-performed filter sets with 4–8 channels in terms of PSNR, GFC, MSE, and CIEDE2000 respectively are computed the same way in Section 4.1.2. The cumulative ranking scores are listed in Table 2, where the best-performing filter set series and the highest cumulative scores are printed in bold.

#### 4.2.2. Comparison between the Best Filer Set and the Traditional Selection by MLI 

The $\;\ell_{2}$ norms for the transmittance vectors of the 45 filters are computed, and the results reveal that that of the No. 2 filter was the maximum. From Table 2, we can see the No. 44 and 45 filter set series have same maximum cumulative score (57), while the No. 44 filter set series has a smaller 6 number of channels comparing with the No. 45 filter set series. Therefore, the No. 44 can be considered as the best-performing filter set series. The evaluative results of the best-performing No. 44 and the No. 2 filter set series with six channels are listed in Table 3 for comparison. From Table 3, we can see the performances are greatly improved comparing No. 44 with No. 2. The same conclusion can also be made from Table 2. As it indicates, all the filter set series listed outperform the No. 2 filter set series, no matter what the numbers of channels are, because the No. 2 filter set series is not in the top 20% filter sets series. 

### 4.3. Characteristics of the Best-Performed Filter Sets

#### 4.3.1. Condition Number

**Figure 5:**
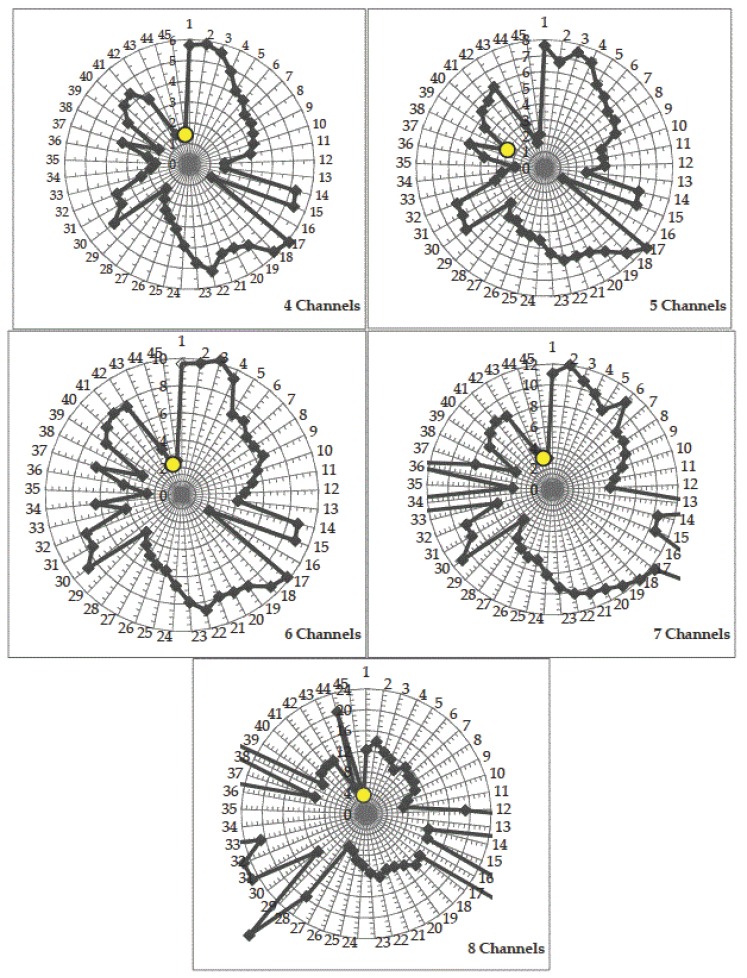
Radar charts of the condition numbers versus the corresponding filter sets in terms of several channel numbers. The radical coordinates denote condition numbers and the angular coordinates denote the No. of the filter sets, therefore the markers denote the position of condition number of the corresponding filter sets. In the radar charts at seven and eight channels, some of the condition numbers cannot be seen because they are too large to be displayed, however only the filter sets with the smaller ones deserve for consideration.

The condition numbers of the best-performing filter set series the No. 2 series are listed in Table 4, where the figures printed in bold are the condition numbers of the filter sets in the best-performing channels. The condition numbers of the entire 45 filter set series are displayed in Figure 5 by radar charts, where the condition numbers of the best-performed filter sets are displayed in bright yellow round marks. From Table 4, we can see the condition numbers of the best filter sets are less than four, although the condition numbers are not always the minimum for the best-performing filter set. From Figure 5, we can see clearly that the condition numbers listed in Table 4 are one of the few smallest among all the condition numbers. Especially, it is more precise when the channel number is not less than 6. Therefore, it indicates that minimizing the condition number of a filter set is an essential precondition for optimizing the sensitivity of broadband camera, and the best-performed filter set must be the one with a smaller condition number than most of the others.

#### 4.3.2. Transmittance Curves 

**Figure 6:**
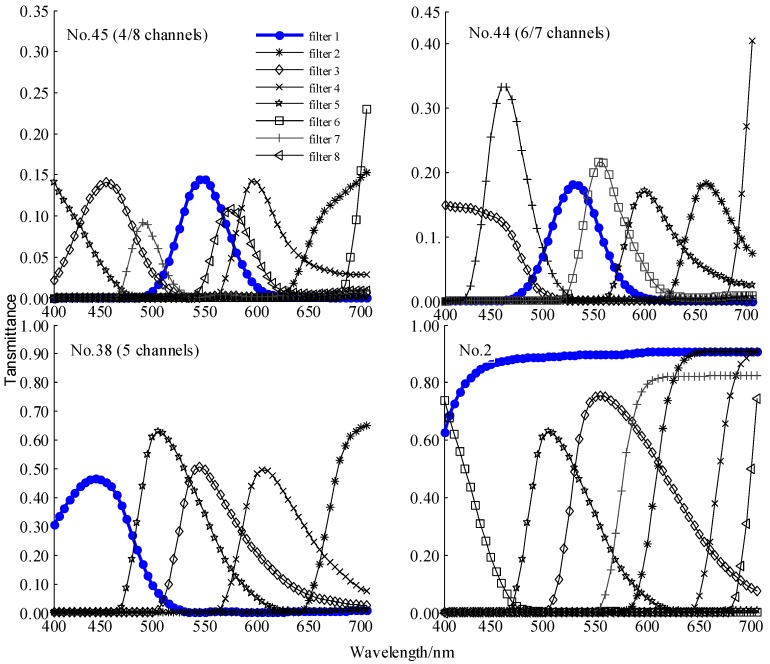
Transmittance curves of the verified best-performed filter sets under CIE standard illuminant D65 at 4, 5, 6, 7and 8 channels (No. 45, 44 and 38) and the filter set series with maximum $\;\ell_{2}$ norm first filter (No. 2), where the first filter of the filter sets is graphed in bold line with solid round markers. The filter sets are selected the same way as the traditional MLI algorithm charted in Section 2 by minimizing condition number at each channel with the designated filter as the first filter of the corresponding filter set. The notation, for example, No. 38 (5 channels) denotes the No. 38 filter set is superior to others when the number of channels is 5, and the No. of the first filter of the filter set is 38.

For further investigation, the transmittance curves of the best-performing filter sets in Table 4 are graphed in Figure 6. Comparing to the No. 2 filter set from Figure 6, either of the first filters of the best-performing filter sets has a distinct transmittance peak and the peaks of the sequential filters in a specific filer set are distributed almost evenly in the wavelength range. Comparing the No. 38 with No. 45 or No. 44 filter sets at five channels, the best-performing filter set (No. 38) has higher peak transmittance and more overlaps than the first five channels of No. 44 or No. 45. Similarly, the distribution of the first four filters of the best-performing No. 45 filter set features the same geometric characteristics comparing with the No. 44 filter set. It is worth noting that there is a large notch in the transmittance curves of the first four filters of the No. 44 filter set at the wavelength of approximately 620 nm, which may be the cause of the No. 44 filter set performing worse than that of the No. 45 filter set at four channels. 

In order to quantify the geometry features of the best-performing filter set, we can define metrics to measure the uniformity of the peaks distribution and the degree of overlaps of a filter set in the wavelength range. If the Gaussian function is adopted, the mean and standard derivation can describe the geometry features of the filter curves of the best-performed filter sets. Unfortunately, the filter set is appropriate to be normalized to fit the Gaussian function under conventional imaging conditions, because it is hard to gain same responses of different channels by modulating the imaging parameters easily. The uniformity of the peaks distribution can be defined as:

(11) $$UF=1-\frac{\left|\sum_{i=1}^{i=M}(V_{pfi-}V_{pri})\right|}{\sqrt{\sum_{i=1}^{i=M}V_{pfi}^{2}+\sum_{i=1}^{i=M}V_{pri}^{2}}},$$

where $V_{pfi}$ and $V_{pri}$ are the *i-*th coordinate in terms of wavelength of the peaks of the filters that are sorted in ascending order and the corresponding ideal reference coordinate; *M* is the number of channels; *UF* = 1 means the peaks of a filter set are perfectly uniformly distributed. The degree of overlaps of a broadband filter set can be described as:

(12) $$OLP=\frac{(\sum_{i=1}^{M}\left(\lambda_{Rthri}-\lambda_{Lthri}\right))-\left(\lambda_{max}-\lambda_{min}\right)}{M\left(\lambda_{max}-\lambda_{min}\right)},$$

where $\lambda_{Rthri}$ and $\lambda_{Lthri}$ are the right and left wavelength thresholds of the bandpass of the *i-*th filter of a filter set; OLP≤0 means there are no overlaps in a broadband filter sets. Note that the *Uniformity* and *Degree of overlaps* defined above is only for quantifying of the geometric features of the best-performed filter sets. In this article, the threshold wavelength adopts the wavelength where 10% of the transmittance of the filter peaks locates. The geometric measurements of the best-performed filter set are listed below in Table 5. The data listed in it may serve as a reference to a physically realizable broadband filter sets by selecting from among commercially available absorption filters.

## 5. Discussion

### 5.1. General Applicability of the MLI Method with Varying Imaging Parameters

The characteristics of the best-performing filter sets are revealed from the above simulation; however, it is significant for their general applicability when changing the imaging parameters. Among the parameters related in Section 2, the imaging illuminant is more uncontrollable than other factors under real conditions, especially the lighting of uncontrollable outdoor spectral imaging [2,26]. Therefore, we make another simulation with the CIE standard illuminant A graphed in Figure 2f. The results corresponding to Table 2 are listed in Table 6 displaying the cumulative scores of the first four best-performed filter sets under CIE standard illuminant A. 

**Figure 7:**
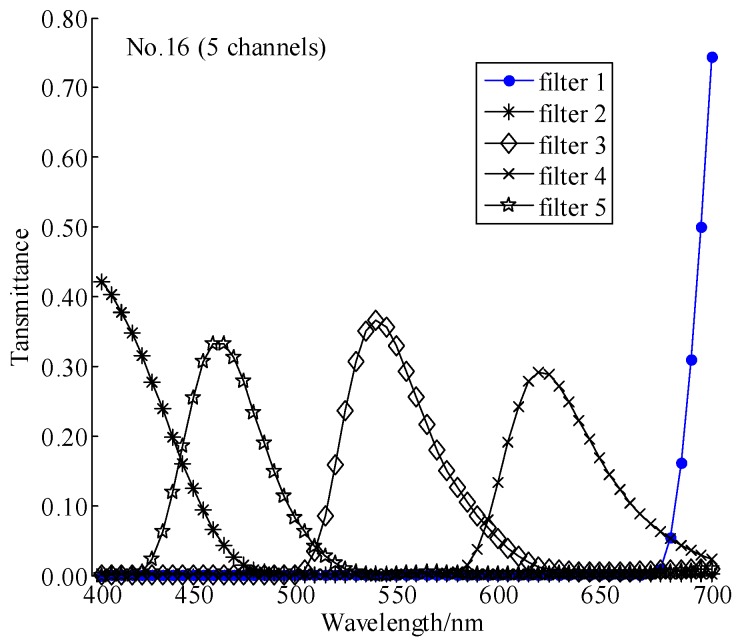
Transmittances of the best-performed filter sets, No. 16 (5), under CIE standard illuminant A according to the cumulative scores listed in Table 6.

Comparing to Table 4, the best-performing filter set in Table 6 are the same at channels 4, 6, 7 and 8, although under a different light source. A difference is the No. 16 filter set at channels 5 from comparison of the cumulative scores listed in Table 6 with 2. The transmittance curves of the No. 16 filter series are displayed in Figure 7, and the condition numbers of the No. 16 filter series are listed in Table 6. Comparing the geometric features of the transmittance curves of the No.16 filter sets in Figure 7 with those of the No. 38 in Figure 6, the same conclusions as in Section 4.3.2 above under CIE standard illuminant D65 can be made, and so do the condition numbers in Table 6. From the cumulative ranking scores (see Table 6), in fact, we can see No. 38 is the closest five channels filter set just behind the best-performing No. 16 filter set. Looking back at Table 2, the scores display the similar way under CIE standard illuminant D65 as displayed in Table 6, in which the first best filter set is No. 38 and the second best one is No. 16. Therefore, the fact that the characteristics of the best-performing filter sets under CIE standard illuminant A supports our conclusion about the criteria to identify the best-performing filter sets derived under CIE standard illuminant D65. In other words, the criteria for selecting a filter set to optimize the spectral sensitivity of broadband camera would be insensitive to varying the imaging parameter, or light source which illuminates the imaging scene. Other imaging parameters may be changed for equivalent simulations such as varying the camera spectral sensitivity function and the spectral imaging scene; however, it is not necessary to do that. The generalization of the conclusions about the criteria of the competent filter set selected by MLI should have been justified by the simulations with varying the light source above. 

### 5.2. Two Intuitive Steps for the MLI Method for Selecting Filter Sets

From the analysis above, we can select the best filter set for optimizing the sensitivity of broadband multispectral camera by the characteristics such as the condition number and the geometric distribution of the transmittance curves. However, the methodology is intuitive and still difficult to operate in practice. The reasons are two-fold. The first is that the best-performing filter set is not always the one with the smallest condition number, as it have been shown in Table 4 and Table 7, and Figure 7. The other is that the characteristics of the transmittance curves of the filter set are too intuitive to be handled quantitatively. Therefore, we recommend two steps to resolve the problem.

The first step is to select a subset of the filter sets with smaller condition number from the entire filter sets. Comparing Table 2 and Table 6 to Figure 5, we can see the best-performing filter set stays among a few filter sets with smaller condition number. Table 8 below lists the sequential numbers of the condition number sorted ascendingly from 45 corresponding condition numbers for the best-performing filter sets. From Table 8, we can see that the condition numbers of the best-performing filter sets are almost the closest to the smallest; the maximum deviation is seen for the No. 38 filter set, of which the condition number is smaller than about 89% of other 40 filter sets. Namely, if the subset of filter sets is composed by the five filter sets with the smallest condition number, we can decide the best-performed filter set is in it.

The second step is to select a filter set among the subset in terms of the geometric distribution of its transmittance curves or by a few experimental explorations. The geometric distribution of the transmittance curves of a best-performing filter set related above would give an intuitive criterion to conveniently identify the best-performing filter set from the subset, otherwise experimental explorations can be conducted with every filter set in the subset to pick out the best-performing filter set according to the experimental results. Because of the number of the filter sets in the subset is less than five according to the typical case in this article, it should be an efficient way to select the best-performing filter set in the subset.

### 5.3. Possible Application and Future Work

Although derived by the simulation with commercial absorption filters illustrated in Figure 1a, the experimental framework of the MLI method enhanced by the work in this article can be applied to other aspects such as transmittance design for SFA multispectral system and direct optimization of physical variables for filter manufacturing techniques. The criteria for selecting filter sets can serve as an explanation of why a broadband multispectral camera with overlapping spectral sensitivities works well for the conventional SFA multispectral system [14,15,16,17,29,30,31], and thus it would be serve as a criterion for designing the spectral sensitivity of SFA sensors. As a practical approach, filters could also be designed using direct optimization of dye concentrations [32], consequently, the criterion could guarantee a desired optimal filter set manufacturable in reality.

It is worth noting that one reason for only selecting 45 single filters from 1035 filters as the first filter is to reduce the calculation pressure; the second is that single filter is easier to implement than a combination of single filters in practical applications. The other 990 filters achieved by combination of two single filters may serve as the first filter, and this would produce more filter set series to be selected by the MLI method related above, therefore it would lead the spectral sensitivity of a broadband multispectral camera to be more optimized.

It is worth noting that the results of the best filter sets are amazing due to the fact their shapes are somehow similar to those of the transmittances curve of the state-of-art SAF, however it does not mean that the MLI method advanced above can be directly applied to SAF design. It would be a good idea to use the scoring or ranking method described in the manuscript to search for the other types of optimal filter sets [14,15,16,17,29,30,31]. In a typical case, the authors try to find five optimal filters of Gaussian curve type by varying five parameters [31]. We can refer to the results displayed in the Figure 4 and the Table 2 in this work, where the PNSRs are less than 40 dB (36 dB on average) in most cases under noise free conditions [31]. While the average PNSR of No. 38 (5), the best-performing filter set at five channels, of the systems in this article (Table 9) is better than the results in [31] comparing by quantities, however, due to the differences of the experimental frameworks, the MLI method using for SFA design would be a future work requiring more investigation. 

## 6. Conclusions

In this paper, we propose a framework of selecting a broadband filter set from a large number of commercial filters according to MLI by imaging simulation. From the results of the simulation, we found the remarkable characteristics of the best-performing filter set. Besides a smaller condition number of the best-performing filter set, the geometric features comprise a distinct peak of the transmittance of the first filter, a generally uniform distribution of the peaks of the transmittance curves of the filter sets, and substantial overlaps of the transmittance curves with those of the adjacent filters. The geometric features of the best-performing filter sets can be quantified by the two metrics, UF and OLP. The proposed method for selecting an optimal filter set guarantees a significant enhancement of the performance of broadband multispectral systems. This work should be useful for optimizing the spectral sensitivity of broadband multispectral imaging sensors.

## Figures and Tables

**Table 1:** Statistics of the top 20% best-performed filter set series, where the No. denotes the No. of filter set series; H-mean denotes descending order of the means; H-min denotes descending order of the minimums; L-mean denotes ascending order of the means and L-min denotes ascending order of the minimums.

**PSNR**				**GFC**			
No.	H-mean	No.	H-min	No.	H-mean	No.	H-min
45	37.68	45	25.10	34	0.9215	34	0.5347
44	35.87	43	21.73	45	0.8829	43	0.0649
43	35.74	11	20.54	43	0.8728	45	0.0193
11	35.55	36	20.22	44	0.8722	11	−0.7395
36	34.78	2	18.56	16	0.8536	41	−0.8475
12	34.75	7	18.04	40	0.7544	44	−0.9224
13	34.49	13	18.00	41	0.7484	40	−1.0018
28	34.34	8	17.95	32	0.7437	36	−1.0851
40	33.83	41	17.87	31	0.7364	16	−1.3993
**MSE**				**CIEDE2000**			
Series No.	L-mean	No.	L-min	Series No.	L-mean	No.	L-min
45	2.20 $\times 10^{-3}$	33	5.68 $\times 10^{-5}$	45	4.67	42	0.09
43	4.43 $\times 10^{-3}$	38	6.27 $\times 10^{-5}$	43	5.13	35	0.10
11	5.06 $\times 10^{-3}$	35	6.33 $\times 10^{-5}$	38	6.88	38	0.11
36	6.14 $\times 10^{-3}$	42	6.33 $\times 10^{-5}$	13	6.94	33	0.13
44	7.84 $\times 10^{-3}$	45	6.40 $\times 10^{-5}$	44	6.98	26	0.14
12	8.22 $\times 10^{-3}$	3	6.41 $\times 10^{-5}$	11	7.04	27	0.14
13	8.69 $\times 10^{-3}$	27	6.48 $\times 10^{-5}$	36	7.15	1	0.14
28	9.66 $\times 10^{-3}$	26	6.49 $\times 10^{-5}$	12	7.67	5	0.15
2	1.08 $\times 10^{-2}$	5	6.52 $\times 10^{-5}$	28	7.77	3	0.15

**Table 2:** Cumulative scores of the nine best-performed filter sets or series under CIE standard illuminant D65.

No.	Scores	No.	Scores	No.	Scores	No.	Scores	No.	Scores	No.	Scores
4 channels		5 channels		6 channels		7 channels		8 channels		all channels	
**45**	43	**38**	48	**44**	**57**	**44**	50	**45**	**57**	**45**	56
43	42	16	42	45	41	35	47	34	31	43	46
38	33	44	39	11	37	38	38	12	31	11	30
16	31	45	31	16	36	45	32	28	29	44	28
36	30	13	30	40	30	43	27	33	28	38	22
13	27	35	20	43	27	34	22	43	28	36	22
29	23	34	18	42	21	33	18	13	24	34	18
35	23	40	14	33	19	25	16	27	23	42	15
44	16	43	13	34	16	27	14	42	15	35	15

**Table 3:** Comparison of the performances between the best-performed filter set No. 44 and the traditional suboptimal selection No. 2 at 6 channels.

Indices	PSNR		GFC		MSE		DE2000	
No.	44	2	44	2	44	2	44	2
50db	45.72	43.30	0.9984	0.9663	2.80 $\times 10^{-4}$	5.62 × 10^−4^	1.05	2.96
40db	40.56	34.37	0.9897	0.8656	5.07 $\times 10^{-4}$	2.3 × 10^−3^	2.30	6.72
30db	32.46	25.53	0.9139	−0.0465	2.80 $\times 10^{-3}$	1.99 × 10^−2^	6.22	16.26

**Table 4:** Comparison of the condition numbers of the best-performed filter set (No. 45, 38 and 44) and the traditional suboptimal selection (No. 2).

Channels	No. 45	No. 38	No. 44	No. 2
4	**1.45**	1.65	1.37	5.84
5	2.11	**2.56**	1.63	6.65
6	2.55	3.21	**2.31**	9.74
7	3.04	3.91	**3.10**	12.01
8	**3.62**	92.35	20.34	13.99

**Table 5:** The geometric measurements of the best-performed filter.

No./Channels	45/4	38/5	44/6	44/7	45/8
***UF***	0.950	0.983	0.937	0.983	0.984
***OLP***	0.094	0.176	0.120	0.142	0.132

**Table 6:** Cumulative scores of the four best-performed filter sets under CIE standard illuminant A.

No.	Scores	No.	Scores	No.	Scores	No.	Scores	No.	Scores
4 channels		5 channels		6 channels		7 channels		8 channels	
**45**	43	**16**	50	**44**	52	**44**	50	**45**	57
43	40	38	42	45	43	35	46	34	31
16	36	45	39	16	38	38	37	12	31
38	33	44	37	34	31	45	31	28	29

**Table 7:** Condition number of the No.16 series.

Channels	4	5	6	7	8
**Cond**	1.22	**1.30**	2.30	22.15	2.01 $\times 10^{-5}$

**Table 8:** The sequential number in ascending order from the 45 condition numbers for the best-performed filter sets under CIE standard illuminant D65 and CIE standard illuminant A.

Channels	4	5	6	7	8
Filter No.	45	38	44	44	45
D65	3	5	2	2	1
Filter No.	45	16	44	44	45
A	3	1	2	2	1

**Table 9:** Performances of No. 38 (5) under D65.

S/N	∞	50	47	43	40	37	33	30	27	23
PNSR	43.2	42.1	41.5	40.3	39.0	37.2	34.3	31.9	29.2	25.6
